# Delineating patterns of sexualized substance use and its association with sexual and mental health outcomes among young gay, bisexual and other men who have sex with men in Singapore: a latent class analysis

**DOI:** 10.1186/s12889-021-11056-5

**Published:** 2021-05-31

**Authors:** Rayner Kay Jin Tan, Caitlin Alsandria O’Hara, Wee Ling Koh, Daniel Le, Avin Tan, Adrian Tyler, Calvin Tan, Chronos Kwok, Sumita Banerjee, Mee Lian Wong

**Affiliations:** 1 Saw Swee Hock School of Public Health, National University of Singapore, 12 Science Drive 2, MD1 Tahir Foundation Building #10-01, Singapore, 117549 Singapore; 2 Yong Loo Lin School of Medicine, National University of Singapore, 10 Medical Dr, Singapore, 117597 Singapore; 3 Action for AIDS Singapore, 9 Kelantan Lane #03-01, Singapore, 208628 Singapore; 4 National University Hospital, National University Health System, Singapore, Singapore

**Keywords:** Chemsex, Alcohol, Poppers, MSM, Singapore

## Abstract

**Background:**

Young gay, bisexual, and other men who have sex with men (YMSM) are vulnerable to the risks associated with sexualized substance use. This is a novel study in Singapore that aims to classify patterns of sexualized substance use among YMSM, and investigate its association with sexual and mental health outcomes.

**Methods:**

In this cross-sectional study among 570 YMSM aged 18 to 25 years old, latent class analysis (LCA) conducted to identify classes with similar patterns of sexualized substance use, across which measures of inconsistent condom use, recent STI diagnoses, past suicide ideation and depression severity were compared.

**Results:**

LCA revealed three classes of YMSM based on types of substances ever used in sexualized contexts, which we labelled as ‘substance-naive’, ‘substance-novice’, and ‘chemsex’. Substance-naive participants (*n* = 404) had only ever used alcohol, while substance-novice participants (*n* = 143) were primarily amyl nitrite users with a small proportion who reported using chemsex-related drugs. Chemsex participants (*n* = 23) comprised individuals who had mostly used such drugs. Those in the chemsex group were more likely to report recent unprotected anal sex with casual partners (aPR = 3.28, 95%CI [1.85, 5.79]), depression severity (aβ = 3.69, 95%CI [0.87, 6.51]) and a history of suicide ideation (aPR = 1.64, 95%CI [1.33, 2.03]).

**Conclusions:**

Findings of this study highlight how the use of varying substances in sexualized contexts may be classified and characterized by different sexual and mental health outcomes. Health promotion efforts should be differentiated accordingly to address the risks associated with sexualized substance use among YMSM.

**Supplementary Information:**

The online version contains supplementary material available at 10.1186/s12889-021-11056-5.

## Background

Approximately 38.0 million people globally were estimated to be living with HIV in 2019 [1]. Gay, bisexual, and other men who have sex with men (GBMSM) constitute a key population that continues to be disproportionately affected by HIV on a global scale [2]. Greater vulnerability of GBMSM to HIV may be attributable to physiological, psychosocial, and institutional factors [3, 4]. For example, past research have shown how measures of sexual orientation concealment, internalized homophobia, as well as experienced homophobia have been associated with greater risks of HIV and other sexually transmitted infections acquisition among GBMSM [5–7].

Past studies have shown that the prevalence of drug use is higher among GBMSM, compared to the general heterosexual male population, and is often used in sexualized contexts [8–10]. Sexualized substance use has also been referred to by other terms such as ‘chill fun’, ‘chemsex’, ‘party and play’ and ‘wired sex’, which vary by country setting. These terms typically denote the use of substances such as mephedrone, amyl nitrites (poppers), gamma-hydroxybutyrate/gamma-butyrolactone (GHB/GBL), crystal methamphetamine (meth), as well as drugs usually prescribed for erectile dysfunction (ED) before or during sexual activity [11–13].

Chemsex has been associated with several health implications among GBMSM. With regards to sexual health, studies have found that GBMSM who have engaged in chemsex were more likely to report HIV and other STI risk-related behaviors, as well as the incidence of HIV and sexually transmitted infections (STIs) [14, 15]. With regard to mental health, GBMSM who engage in chemsex have also reported correspondingly poorer mental health outcomes, such as greater depression severity, poorer general mental well-being, and internalized homophobia [13, 16, 17]. Heavy alcohol use has also been found to be common among GBMSM in developed country settings [9, 18, 19], and that sexual minorities such as GBMSM reported a lower age of alcohol use debut compared to their heterosexual counterparts [20, 21]. Such early onset of alcohol use was associated with increase sexual and mental health risks among GBMSM surveyed [22]. The use of alcohol in a sexual context has also been found to be associated with behaviors associated with HIV acquisition risk [23–25].

Within communities of GBMSM, young gay, bisexual, and other men who have sex with men (YMSM) experience a greater burden of HIV and other sexually transmitted infections (STI) risk compared to their older counterparts, which may be attributed to a higher incidence of risky sexual behaviors, the lack of help-seeking behaviors, and sexualized substance use [26, 27]. YMSM are also especially vulnerable to substance use disorders and the risks associated with them [28–30]. A study among YMSM aged 12 to 24 years in eight United States cities found that 10.8% of participants had reported using methamphetamine in the past 3 months [28], while studies in other settings similarly illustrate that YMSM already report using illicit substances at a young age [13, 31], and those who report an earlier age of sexual debut or who are exposed to sexual networks earlier are more likely to initiate chemsex [32].

Past studies employing latent class analysis have found that substance use patterns may not be homogenous among GBMSM, and these classes are characterized by a range of substance use-related behaviors, from GBMSM who do not use substances in sexualized contexts, to those who engage in polydrug use during sex. These studies also find that these classes may be associated with varying sexual risk behaviors [14, 33]. Similarly, the present study will conduct latent class analysis on observational, cross-sectional data from the baseline analysis of the Pink Carpet Y Cohort Study (PCYCS), Singapore’s first prospective cohort study among YMSM, to explore classes of sexualized substance use and its association with other sexual and mental health outcomes. This study is noteworthy as it is novel in the present setting, and allows us to better understand the risk factors that may be associated with substance use initiation among YMSM.

## Methods

### Country setting

GBMSM are disproportionately represented in prevalent cases of HIV in Singapore. As of 2019, a total of 8295 incident HIV infections among Singapore residents have been notified to the Singapore ministry of health (MOH). In 2011, yearly incident cases of HIV transmitted through male ‘homosexual or bisexual’ modes exceeded that of ‘heterosexual’ modes for the first time, and that trend has persisted since [34]. With regard to GBMSM in Singapore, the general public still holds conservative and largely negative views towards the community. Specifically, most people have indicated in recent surveys that they had perceived same-sex relationships as being wrong, and are also not in favor of the repeal of Section 377A of the Singapore Penal Code, the law that criminalizes sexual relations between men [35, 36]. Past studies have also established the negative impact and trickle-down effects that such stigma has on HIV prevention efforts among GBMSM in Singapore [37–39]. With regard to substance use, a qualitative study conducted on chemsex among GBMSM in Singapore highlight how institutionalized and societal stigma contributed to internalized homophobia that drive chemsex as a coping mechanism, as well as barriers to accessing substance use recovery and care services [13].

Advocacy and health promotion efforts for GBMSM in Singapore have largely stemmed from community-based efforts, given the presence of Section 377A, which precludes the development and implementation of wider programs such as comprehensive sexuality education and GBMSM-specific healthcare services, or the censorship of positive portrayals of LGBT individuals in the general media [40]. Such community-based efforts include the implementation of community-based anonymous testing sites for HIV and other STIs, as well as HIV and other STIs prevention efforts around education, promoting sexual health-seeking behaviors, and the uptake of novel HIV prevention technologies such as HIV pre-exposure prophylaxis and post-exposure prophylaxis in key populations at risk of acquiring HIV and other STIs [41].

### Participants and recruitment

The PCYCS is a prospective cohort study exploring the syndemic risks associated with HIV and other sexually transmitted infections (STI) acquisition among YMSM in Singapore. However, this study presents analysis from observational, cross-sectional data obtained from the baseline survey of the PCYCS. This study was a partnership between Action for AIDS Singapore (AFA), an organization serving the sexual health needs of GBMSM, and the National University of Singapore (NUS). To be eligible for this cohort, participants had to be HIV-negative or unsure of their HIV status, between the ages of 18 to 25 years old, Singapore citizens or permanent residents, and identify as gay, bisexual, or queer (sexual orientation) men at the point of recruitment, which spanned across May to September 2019. Participants were asked to self-report these attributes. With regard to sample size calculation, we targeted recruitment of at least 384 participants. This sample size was obtained to achieve a 95% confidence level and 5% margin of error, assuming a population of 210,000 GBMSM in Singapore [42]. However, 600 participants were targeted for recruitment to account for potential attrition at each follow-up for this cohort study.

Participants were invited to participate in this study through a recruitment flyer that was disseminated through both online (e.g. social media) and offline (e.g. at the organization’s office or outreach activities) channels by a network of community-based organizations in Singapore who are engaged in health advocacy-related activities for GBMSM. Participants who were interested in participating and were eligible for the study signed up through an enrolment link with their self-reported alias, contact details, date of birth, gender, HIV status, sexual orientation, and their residence status. An AFA staff member subsequently verified the eligibility of participants who had signed up prior to sending them a unique identifier, and a link for the baseline survey.

It was imperative for the team to ensure that participants’ identities their data would remain confidential, as drug use and sexual relations between men are criminalized in Singapore. To do so, the researchers ensured that no staff member from AFA or NUS had full access to either the enrolment details held by AFA which contained aliases and contact details of participants, and the baseline survey results held by NUS. Both sets of data were only linked by the unique identifier which participants entered at the beginning of the survey. Upon completion of the survey, a NUS staff member provided AFA with the unique identifiers who had completed the baseline survey, and an SGD20.00 (approximately USD15.00) cash reimbursement was given to the participant. A total of 570 participants were recruited at the baseline of the cohort; the response rate could not be established as it was not possible to ascertain the total number of eligible participants that the recruitment flyers had reached. Participants could also refer their friends to participate in the survey and be reimbursed SGD5.00 (approximately USD3.75) for each eligible individual successfully referred and who had completed the baseline survey; a total of 171 (30.0%) of participants were recruited through referrals.

### Ethics declaration

Ethics approval was obtained from the institutional review board at the National University of Singapore (NUS-IRB Reference Code S-19-007) prior to data collection.

### Variable measures

A copy of the survey questionnaire developed for this study has been published elsewhere [43]. The survey collected sociodemographic information from respondents, including age, ethnicity, gender, sexual orientation, and monthly household income. Participants were asked if they had ever used a series of substances in sexual contexts, including alcohol, poppers, meth, GHB/GBL as well as other erectile dysfunction (ED) medication or drugs (e.g. Viagra, Cialis, ‘black ants’). For sexual health outcomes, participants were asked about their patterns of unprotected anal sex, as well as STI diagnoses in the last 6 months. Unprotected anal sex with casual partners in the last 6 months was coded as a binary variable (yes vs no), and was derived from a series of questions that solicited frequency of self-reported condom use through a five-point Likert scale from 1 to 5, with 1 being that they did not use condoms and 5 being that they had always used condoms; this question was repeated for permutations of oral and anal sex with regular, casual, and sex worker partners in the last 6 months. Participants who had not used condoms all the time with casual and sex worker partners in the last 6 months were coded as ‘yes’ under this variable. Participants who also reported being tested positive for either gonorrhoea, syphilis, chlamydia, genital herpes, genital warts or hepatitis C were assigned as having been diagnosed with an STI in the past 6 months through a binary (yes vs no) variable.

For mental health outcomes, both depression severity and past suicide ideation were measured. Depression severity, which was measured through the well-established, nine-item patient health questionnaire-9 (PHQ-9) validated by Kroenke and colleagues [44, 45]. Participants were asked “over the last 2 weeks, how often have you been bothered by any of the following problems?” to a total of nine statements, to which they could respond to four possible answers on a Likert scale; 1 being *not at all* and 4 being *nearly every* day. Depression severity was measured as an index that was the sum score of all nine items, with a minimum score of 0 and a maximum score of 27. A score of 20 or higher indicates severe depression, 15 to 19 suggests moderately severe depression, 10 to 14 suggests moderate depression, while a score between 5 to 9 indicates mild symptoms of depression. Cronbach’s alpha of the scale was reported as 0.92. Participants were also asked about their suicide-related behaviors, including if they had ever contemplated suicide by responding to three possible answers: *yes, no,* or *prefer not to say*.

### Statistical analysis

Statistical analysis was carried out using the statistical software STATA version 15 (Stata Corp, College Station, TX, USA). We employed descriptive statistics to identify trends in sample characteristics. Details on the latent class analysis employed has been published elsewhere [32], where the chosen variables included a history of using alcohol, poppers, meth, GHB/GBL as well as other ED medication or drugs (e.g. Viagra, Cialis, ‘black ants’) in sexualized contexts. Given that the three-class model had the lowest AIC and BIC values, it was thus reported in this study, which we labelled post hoc as ‘substance-naïve’, ‘substance-novice’ and ‘chemsex’. A summary of goodness-of-fit statistics are provided in Supplementary Table S1. Following identification of latent classes, we sought to determine the association between varying classes with outcome variables of unprotected anal sex, STI diagnoses, depression severity and past suicide ideation while adjusting for key sociodemographic covariates. We employed multivariable Poisson regression models with robust sandwich variances to compute the crude prevalence ratio (PR) and adjusted prevalence ratio (aPR) estimating these outcome variables. Statistical significance was set at *p* < 0.05. Analysis for this study was not pre-registered and the results reported here should be considered exploratory.

## Results

### Sociodemographic attributes and description of analytic sample

A total of 570 participants were recruited in this study. Table 1 summarizes the sociodemographic attributes and overall description of the analytic sample. In terms of their sociodemographic attributes, the mean age of the sample was 21.9 years (SD = 2.17). 83.9% of the participants identified as Chinese (*n* = 478), 92.1% identified as cisgender male (*n* = 525), 71.6% identified as gay (*n* = 408), and 35.6% reported a monthly household income of SGD5000 and above (*n* = 203). A total of 33.5% (*n* = 189), 28.0% (*n* = 158), 4.6% (*n* = 26), 4.6% (*n* = 26), and 4.6% (*n* = 26) reported ever using alcohol, poppers, meth, GHB/GBL, and ED medication or drugs in sexual contexts, respectively.

**Table 1:** Description of analytic sample (*n* = 570)

Variables	n / Mean	% / SD
**Age**	21.9	2.17
**Chinese ethnicity (Ref = Non-Chinese)**	478	83.9%
**Cisgender male (Ref = Transgender, genderqueer, or others)**	525	92.1%
**Gay (Ref = Bisexual, queer, or others)**	408	71.6%
**Monthly household income ≥ SGD5000 (Ref < SGD5000)**	203	35.6%
**Ever had sexualized substance use with**		
Alcohol	190	33.3%
Amyl nitrites (Poppers)	161	28.3%
Crystal methamphetamine (Meth)	27	4.7%
Gamma-hydroxybutyrate/gamma-butyrolactone (GHB/GBL)	27	4.7%
Erectile dysfunction drugs (ED)	26	4.6%
**Ever contemplated suicide**	308	54.0%
**Unprotected anal sex with casual partners in the last 6 months**	96	16.8%
**Tested positive for an STI in the last 6 months**	39	6.8%
**Depression severity (PHQ-9)**	7.9	6.79

*Abbreviation*: *SD* Standard Deviation, *STI* Sexually Transmitted Infections, *GHB/GBL* Gamma-Hydroxybutyrate/Gamma-Butyrolactone, *PHQ-9* Patient Health Questionnaire-9

With regard to the outcome variables of the study, participants reported a mean score of 7.9 (SD = 6.79) on depression severity, while most participants had ever contemplated suicide (*n* = 308, 54.1%). A total of 16.8% of participants also reported unprotected anal sex with casual partners in the last 6 months (*n* = 96), while 6.7% (*n* = 38) reported testing positive for an STI in the past 6 months.

### Patterns of sexualized substance use

**Fig. 1:**
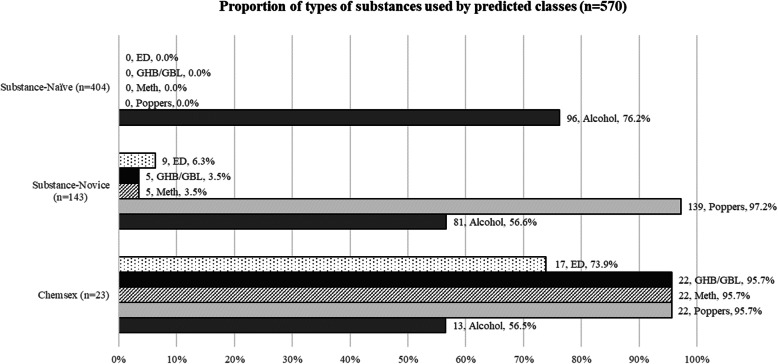
Proportion of types of substances used by predicted classes (*n*=570)

LCA revealed three classes (AIC = 1743.14; BIC = 1808.33), which we labelled post hoc as ‘Substance-naive’, ‘Substance-novice’, and ‘Chemsex’. Participants in the substance-naive (*n* = 403) class reported only ever using alcohol (*n* = 96, 76.2%), and never using any other substances during sex. Participants in the substance-novice class (*n* = 143) had mostly ever used poppers during sex (*n* = 139, 97.2%), with some reporting ever using meth (*n* = 5, 3.9%), GHB/GBL (*n* = 5, 3.9%), and erectile dysfunction drugs (*n* = 9, 6.3%) during sex, over and above those who reported ever using alcohol during sex (*n* = 81, 56.6%). A similar proportion of participants in the chemsex class (*n* = 23) reported ever using alcohol (*n* = 13, 56.5%) and poppers during sex (*n* = 22, 95.7%), but a large proportion reported ever using meth (*n* = 22, 95.7%), GHB/GBL (*n* = 22, 95.7%), and erectile dysfunction drugs (*n* = 17, 73.9%) during sex. Table 2 describes the analytic sample by substance using classes, while Fig. 1 summarizes the proportion of individuals who reported ever using varying substances by substance using classes.

**Table 2:** Description of analytic sample by predicted substance-using classes (*n* = 570)

Variables	Substance-Naïve (***n*** = 404)		Substance-Novice (***n*** = 143)		Chemsex (***n*** = 23)		Fisher’s Exact Test ***p***-value
	n/Mean	%/SD	n/Mean	%/SD	n/Mean	%/SD	
**Age**	21.7	2.18	22.4	2.11	22.3	1.77	0.001
**Chinese ethnicity (Ref = Non-Chinese)**	336	83.2%	124	86.7%	18	78.3%	0.436
**Cisgender male (Ref = Transgender, genderqueer, or others)**	365	90.4%	139	97.2%	21	91.3%	0.018
**Gay (Ref = Bisexual, queer, or others)**	268	66.3%	122	85.3%	18	78.3%	< 0.001
**Monthly household income ≥ SGD5000 (Ref < SGD5000)**	131	32.4%	65	45.5%	7	30.4%	0.019
**Ever had sexualized substance use with**							
Alcohol	96	76.2%	81	56.6%	13	56.5%	< 0.001
Amyl nitrites (Poppers)	0	0.0%	139	97.2%	22	95.7%	< 0.001
Crystal methamphetamine (Meth)	0	0.0%	5	3.5%	22	95.7%	< 0.001
Gamma-Hydroxybutyrate/Gamma-Butyrolactone (GHB/GBL)	0	0.0%	5	3.5%	22	95.7%	< 0.001
Erectile dysfunction drugs (ED)	0	0.0%	9	6.3%	17	73.9%	< 0.001
**Ever contemplated suicide**	207	51.2%	82	57.3%	19	82.6%	0.007
**Unprotected anal sex with casual partners in the last 6 months**	51	12.6%	36	25.2%	9	39.1%	< 0.001
**Tested positive for an STI in the last 6 months**	22	5.5%	15	10.5%	2	8.7%	0.083
**Depression severity (PHQ-9)**	7.9	6.69	7.4	6.60	11.4	8.87	0.030

*Abbreviation*: *SD* Standard Deviation, *STI* Sexually Transmitted Infections, *GHB/GBL* Gamma-Hydroxybutyrate/Gamma-Butyrolactone, *PHQ-9* Patient Health Questionnaire-9

### Association between substance use class membership and sexual health outcomes

Table 3 summarizes the associations between substance use class membership and sexual health outcomes. At the bivariate level, participants who were in the substance-novice (PR = 1.99, 95%CI [1.36, 2.92]) and chemsex (PR = 3.10, 95%CI [1.75, 5.49]) classes were more likely than substance-naive participants to have reported engaging in unprotected anal sex with a casual partner in the last 6 months. Participants who were in the substance-novice class were also more likely that those in the substance-naive class to have reported a diagnosis for any STI in the last 6 months (PR = 1.93, 95%CI [1.03, 3.61]). At the multivariable level, analyses revealed that an increasing age was negatively associated with unprotected anal sex with casual partners in the last 6 months (aPR = 0.91, 95%CI [0.84, 0.99]), while participants who were in the substance-novice (aPR = 2.24, 95%CI [1.51, 3.33]) and chemsex (aPR = 3.28, 95%CI [1.85, 5.79]) classes were more likely than substance-naive participants to have reported engaging in unprotected anal sex with a casual partner in the last 6 months. Participants who were in the substance-novice class were also more likely that those in the substance-naive class to have reported a diagnosis for any STI in the last 6 months (aPR = 2.01, 95%CI [1.05, 3.85]).

**Table 3:** Multivariable Poisson regression for sexual health outcomes and its association with substance using classes

	Unprotected anal sex with casual partners in the last six months (***n*** = 96)				STI diagnoses in the last six months (***n*** = 39)			
	PR	95% CI	aPR	95% CI	PR	95% CI	aPR	95% CI
**Age**	0.94	(0.87, 1.02)	**0.91**	**(0.84, 0.99)**	0.95	(0.83, 1.08)	0.92	(0.80, 1.06)
**Chinese ethnicity (Ref = Non-Chinese)**	0.83	(0.53, 1.32)	0.85	(0.53, 1.36)	0.75	(0.35, 1.57)	0.72	(0.35, 1.51)
**Cisgender male (Ref = Transgender, genderqueer, or others)**	0.83	(0.45, 1.53)	0.77	(0.43, 1.37)	1.59	(0.39, 6.37)	1.42	(0.36, 5.63)
**Gay (Ref = Bisexual, queer, or others)**	1.13	(0.74, 1.71)	1.04	(0.67, 1.60)	1.32	(0.64, 2.73)	1.19	(0.57, 2.46)
**Monthly household income ≥ SGD5000 (Ref < SGD5000)**	0.82	(0.55, 1.22)	0.79	(0.52, 1.19)	0.90	(0.47, 1.72)	0.88	(0.46, 1.67)
**Classes**								
Substance-naïve	Ref	Ref	Ref	Ref	Ref	Ref	Ref	Ref
Substance-novice	**1.99**	**(1.36, 2.92)**	**2.24**	**(1.51, 3.33)**	**1.93**	**(1.03, 3.61)**	**2.01**	**(1.05, 3.85)**
Chemsex	**3.10**	**(1.75, 5.49)**	**3.28**	**(1.85, 5.79)**	1.60	(0.40, 6.39)	1.64	(0.42, 6.38)

Notes

*Abbreviations*: *CI* Confidence Interval, *PR* Prevalence Ratio, *aPR* Adjusted Prevalence Ratio

Statistically significant (*p* < 0.05) are highlighted in bold font

### Association between substance use class membership and mental health outcomes

Table 4 summarizes the associations between substance use class membership and mental health outcomes. At the bivariate level, increasing age was positively associated with ever contemplating suicide (PR = 0.96, 95%CI [0.93, 1.00]). Participants who were in the chemsex class (PR = 1.61, 95%CI [1.31, 1.99]) were more likely than substance-naive participants to have reported ever contemplating suicide. Increasing age (β = − 0.41, 95%CI [− 0.66, − 0.15]), being of Chinese ethnicity (β = − 1.92, 95%CI [− 3.43, − 0.40]), and identifying as a cisgender male (β = − 2.48, 95%CI [− 4.55, − 0.42]) were negatively associated with depression severity. Participants who were in the chemsex class were also more likely that those in the substance-naive class to have reported higher scores for depression severity (β = 3.51, 95%CI [0.66, 6.36]). All factors that were statistically significant at the bivariate level remain significant at the multivariable level. Increasing age was positively associated with ever contemplating suicide (aPR = 0.96, 95%CI [0.93, 0.99]). Participants who were in the chemsex class (aPR = 1.64, 95%CI [1.33, 2.03]) were more likely than substance-naive participants to have reported ever contemplating suicide. Increasing age (aβ = − 0.39, 95%CI [− 0.65, − 0.14]), being of Chinese ethnicity (aβ = − 1.86, 95%CI [− 3.37, − 0.34]), and identifying as a cisgender male (a β = − 2.13, 95%CI [− 4.22, − 0.04]) were negatively associated with depression severity. Participants who were in the chemsex class were also more likely that those in the substance-naive class to have reported higher scores for depression severity (aβ = 3.69, 95%CI [0.87, 6.51]).

**Table 4:** Multivariable Poisson regression for mental health outcomes and its association with substance using classes

	Ever contemplated suicide (***n*** = 308)				Depression severity			
	PR	95% CI	aPR	95% CI	β	95% CI	aβ	95% CI
**Age**	**0.96**	**(0.93, 1.00)**	**0.96**	**(0.93, 0.99)**	**−0.41**	**(−0.66, −0.15)**	**−0.39**	**(−0.65, −0.14)**
**Chinese ethnicity (Ref = Non-Chinese)**	0.87	(0.72, 1.04)	0.87	(0.72, 1.04)	**−1.92**	**(−3.43, − 0.40)**	**−1.86**	**(−3.37, − 0.34)**
**Cisgender male (Ref = Transgender, genderqueer, or others)**	0.89	(0.69, 1.15)	0.88	(0.69, 1.14)	**−2.48**	**(−4.55, − 0.42)**	**−2.13**	**(−4.22, − 0.04)**
**Gay (Ref = Bisexual, queer, or others)**	1.04	(0.88, 1.24)	1.06	(0.89, 1.26)	−0.55	(−1.7, − 0.69)	0.04	(− 1.22, 1.31)
**Monthly household income ≥ SGD5000 (Ref < SGD5000)**	1.00	(0.86, 1.18)	1.02	(0.87, 1.20)	0.33	(− 0.83, 1.50)	0.69	(− 0.48, 1.86)
**Classes**								
Substance-naïve	Ref	Ref	Ref	Ref	Ref	Ref	Ref	Ref
Substance-novice	1.12	(0.94, 1.33)	1.16	(0.97, 1.38)	−0.52	(−1.81, 0.78)	− 0.11	(− 1.43, 1.21)
Chemsex	**1.61**	**(1.31, 1.99)**	**1.64**	**(1.33, 2.03)**	**3.51**	**(0.66, 6.36)**	**3.69**	**(0.87, 6.51)**

Notes

*Abbreviations*: *CI* Confidence Interval, *PR* Prevalence Ratio, *aPR* Adjusted Prevalence Ratio, *β* Unadjusted Coefficient, *aβ* Adjusted Coefficient

Statistically significant (*p* < 0.05) are highlighted in bold font

## Discussion

This study sought to identify classes of YMSM based on the substances that they had ever used in sexualized contexts. We explored the sociodemographic and substance use patterns across each of the different classes, and how these classes differed based on their associations with a variety of sexual and mental health outcomes, specifically recent unprotected anal sex with casual partners and STI diagnoses, as well as depression severity and ever contemplating suicide. This study is noteworthy in that, to our knowledge, no prior study has sought to delineate classes of substance use specifically in younger samples of MSM, which allows us to better understand the risk factors that may be associated with substance use initiation. Furthermore, no other study had ever sought to investigate such patterns of substance use among GBMSM in Singapore.

Results of latent class analysis revealed three classes of YMSM based on the substances that they had ever used in sexualized contexts, which we labelled post hoc as ‘*substance-naive*’, ‘*substance novice*’, and ‘*chemsex*’. Those in the substance-naive group had only ever used alcohol, while the substance-novice group were primarily poppers users with a small proportion who reported using drugs typically associated with chemsex, including meth, GHB/GBL, as well as erectile dysfunction drugs [13, 46]. The chemsex group comprised individuals who had mostly used such drugs. This finding aligns with that of other studies, which identified classes of GBMSM characterized by those who were negligible or non-users of recreational substances excluding alcohol, those who were ‘soft’ drug users, and those who were ‘hard’ drug or polydrug users [33]. Several studies have also found find greater nuances in such patterns of use, separating those who are polydrug users without meth or mephedrone, and those who do [14, 47].

Controlling for potential confounding variables, we found that being in the substance-novice or chemsex classes were associated with poorer sexual and mental health outcomes, compared to those who were in the substance-naive class. Given that those in the chemsex class reported both historical and recent indicators of poorer mental health, we believe that this may underpin certain mechanisms that lead to both sexual risk and substance use risk-related behaviors. This corroborates the findings of other studies [4, 48]. As we are not able to establish causation due to the study design, an alternative explanation would be that substance use itself may be a factor that may cause poorer mental health, and disinhibition associated with sexual risk-related behaviors, which corroborate the findings of other studies as well [49]. These two may mechanisms may also be working in tandem to exacerbate poorer sexual and mental health outcomes in the chemsex class, compared to the substance-naive class of YMSM. We remain mindful that we cannot draw a direct association between substance use and some of these outcomes as the time frame assessed for both differ.

Our study has several strengths. As it was conducted among self-identified HIV-negative YMSM aged 18 to 25 years old, we may be identifying certain risk factors and substance use patterns that may place them at risk of HIV and other STI acquisition. We may also draw conclusions around factors that may be associated with substance use initiation without stronger confounds of age, as GBMSM are more likely to be exposed to various substances throughout their life course due to its cumulative availability via sexual and social networks [50].

Our study has several limitations. First, we might expect that our study participants comprising HIV-negative GBMSM would report a lower prevalence of substance, given that measures of substance use have been positively associated with an HIV-positive status among GBMSM in past studies [51, 52]. As such, a similar study involving GBMSM living with HIV is warranted. Second, as HIV status was self-reported in this study, there may be misclassification of HIV-positive individuals as HIV-negative participants in our study. As past studies have shown that sexualized substance use is a risk factor for HIV acquisition [53, 54], such misclassification might have led to a higher prevalence of substance use in our sample than should be expected from a sample of HIV-negative GBMSM. Third, the implementation of a referral system to recruit study participants from existing members of the study cohort may have led to selection effects or referral bias. Unfortunately, no reference data on population level characteristics for GBMSM is Singapore are available for the construction of sample weights, and thus such bias could have led to either and underestimation or overestimation of the effects reported in this study. Fourth, drug use carries severe penalties in Singapore under the Misuse of Drugs Act, which criminalizes the possession and use of drugs with penalties that range from fines of up to S$20,000 to a maximum of 10 years in prison, and trafficking of drugs beyond stipulated thresholds with a mandatory death penalty or life imprisonment. As such, participants may not be entirely honest with their answers around drug use, which may have led to an underreporting of substance use in the present sample. This form of non-differential misclassification would have biased our results towards the null, when in fact the associations between substance use and the outcomes of interest would have been stronger.

We have several recommendations for health promotion and policy interventions. Overall, such health promotion efforts and interventions should acknowledge that substance use-related support needs among YMSM and GBMSM may be heterogenous, and would require a combination of upstream interventions that promote literacy around the risks associated with substance use, and downstream ones that mitigate such risks or promote the well-being of those who require long-term support. For example, policy changes and campaigns should focus on reducing homophobia towards GBMSM and YMSM to improve mental health outcomes, as well as reduce the demand for chemsex as a means of coping with such forms of stigma. Specifically, this may involve the decriminalization of same-sex sexual relations among GBMSM and the implementation of comprehensive sexuality education in schools.

Findings of this study indicate that efforts for early intervention and education for YMSM should focus on reducing the harms associated with engaging in chemsex. Specifically, interventions may be implemented to identify YMSM who may be at risk of the harms associated with chemsex or mental health comorbidities, based on either their substance use patterns or mental health risk, and engage them in holistic care. Engaging such YMSM would provide opportunities for harm reduction efforts around dealing with stigma and poor mental health, alongside HIV and other STIs acquisition as well. Public health communication and campaigns could also work to frame substance use among GBMSM and YMSM as mental health challenges, rather than criminalized and stigmatized behaviors, given its association with stigma and mental health comorbidities.

## Supplementary Information

**Additional file 1: Table S1.** Summary goodness of fit statistics for class membership comparison.

## Data Availability

The datasets used and/or analysed during the current study are available from the corresponding author on reasonable request.

## Abbreviations

GBMSM: Gay, Bisexual and other Men who have Sex with Men
YMSM: Young Men who have Sex with Men
STI: Sexually Transmitted Infections
GHB/GBL: Gamma-Hydroxybutyrate/Gamma-Butyrolactone
AFA: Action for AIDS Singapore
NUS: National University of Singapore
SGD: Singapore Dollars
GHB/GBL: Gamma Hydroxybutyrate and Gamma-Butyrolactone
C: Coefficient
PR: Prevalence Ratio
