# Supplementary material for: Delineating patterns of sexualized substance use and its association with sexual and mental health outcomes among young gay, bisexual and other men who have sex with men in Singapore: a latent class analysis
Source: BMC Public Health. 2021 May 31;21:1026. doi: 10.1186/s12889-021-11056-5 (PMC8166008; doi:10.1186/s12889-021-11056-5)
Supplement: Supplementary file 1 — Additional file 1: Table S1. Summary goodness of fit statistics for class membership comparison. [file 12889_2021_11056_MOESM1_ESM.docx]

| **Table S1. Summary goodness of fit statistics for class membership comparison** | | | | |
| --- | --- | --- | --- | --- |
| **Number of Classes** | **G2** | **df** | **AIC** | **BIC** |
| **2** | 81.59 | 11 | 1799.44 | 1847.24 |
| **3** | 17.29 | 17 | 1747.14 | 1821.02 |
| **4** | 17.29 | 22 | 1757.14 | 1852.75 |
| **5** | 10.00 | 28.00 | 1761.86 | 1883.54 |
|  |  |  |  |  |
| Abbreviation: G2, Likelihood Ratio Test; df, degrees of freedom; AIC, Akaike Information Criterion; BIC, Bayesian Information Criterion | | | | |
